# Association of troponin-defined myocardial injury with adverse long-term survival among patients with chronic kidney disease

**DOI:** 10.1371/journal.pone.0354873

**Published:** 2026-07-30

**Authors:** Fei Ma, Enhong Han, Jianjun Gao

**Affiliations:** 1 Blood Purification Center, Chifeng Municipal Hospital, Chifeng, China; 2 Department of Nephrology, Ninth Medical Center of Chinese PLA General Hospital, Beijing, China; Saud Al-Babtain Cardiac Centre, SAUDI ARABIA

## Abstract

**Objective:**

Chronic kidney disease (CKD) is associated with increased mortality, however, the impact of troponin-defined myocardial injury within this population remains poorly understood. This study aimed to investigate the associations between troponin-defined myocardial injury and long-term mortality in CKD patients.

**Methods:**

This observational study analyzed 22,772,953 weighted records of adult CKD patients from National Health and Nutrition Examination Survey (1999–2004) databases. Myocardial injury was defined by at least one elevated high-sensitivity troponin (hs-cTn) assay, present in 26.6% of the cohort. Cox regression models adjusted for baseline characteristics and comorbidities were used to assess the associations between troponin-defined myocardial injury and all-cause and cardiovascular mortality. Sensitivity analyses excluding patients with known cardiovascular disease (CVD) were performed to evaluate the robustness of the findings.

**Results:**

Patients with troponin-defined myocardial injury were older and had a higher prevalence of CVD, hypertension, diabetes, as well as a lower estimated glomerular filtration rate (eGFR), compared with those without troponin-defined myocardial injury. Over a median follow-up of 11.6 years, survival was significantly worse among patients with troponin-defined myocardial injury at 1, 5, 10, and 15 years. The adjusted hazard ratios (aHR) for all-cause mortality and cardiovascular mortality in patients with troponin-defined myocardial injury were 1.81 (95% CI 1.51–2.17) and 2.03 (95% CI 1.47–2.79), respectively. Sensitivity analysis excluding records with pre-existing CVD showed similar trends, with an aHR of 1.86 (95% CI 1.56–2.21) for all-cause mortality and 2.44 (95% CI 1.83–3.24) for cardiovascular mortality.

**Conclusion:**

As a marker for troponin-defined myocardial injury, hs-cTns were independently associated with worse long-term survival among CKD patients. However, the observational design precludes causal inference, and single time-point troponin measurements limit the assessment of dynamic changes in myocardial injury.

## Introduction

Chronic kidney disease (CKD) is a significant global public health concern, affecting millions of individuals worldwide and leading to high rates of morbidity and mortality, particularly from cardiovascular disease (CVD) [1–4]. Cardiac troponins have gained particular attention in clinical practice due to their high sensitivity and specificity in detecting myocardial injury. Elevated troponin levels, even in the absence of acute coronary syndrome, have been associated with increased cardiovascular morbidity and mortality in the general population [5,6]. In patients with CKD, troponin elevation is commonly observed and has been linked to adverse cardiovascular outcomes, with or without current acute coronary syndrome [7,8]. However, the exact mechanisms underlying troponin elevation in CKD are not fully understood and may involve both myocardial injury and reduced renal clearance of the biomarker. Previous studies have demonstrated that troponin levels can predict short-term cardiovascular events in CKD patients [9–11]. However, only two studies have explored the association between troponin levels and long-term outcomes in this population. One investigation, with a median follow-up of 12.5 years, reported that the risk ratio for cardiovascular death or events associated with elevated high-sensitivity troponin T (hs-cTn T) was greater in patients with CKD than in those without CKD [12]. Similarly, an 8.5-year observational study found that baseline hs-cTn T levels were independently associated with all-cause mortality in patients with CKD [13]. Despite these findings, the association of both hs-cTn T and high-sensitivity troponin I (hs-cTn I), the prognostic implications of biomarker-defined troponin elevations as a phenotype, with all-cause and cardiovascular mortality specifically in the CKD population remain inadequately characterized and warrant further investigation. The aim of this study was to investigate the association between myocardial injury, as indicated by high-sensitivity troponin (hs-cTn) assays, and long-term survival outcomes in a large cohort of patients with CKD.

## Methods

### Study design and population

**Fig 1:**
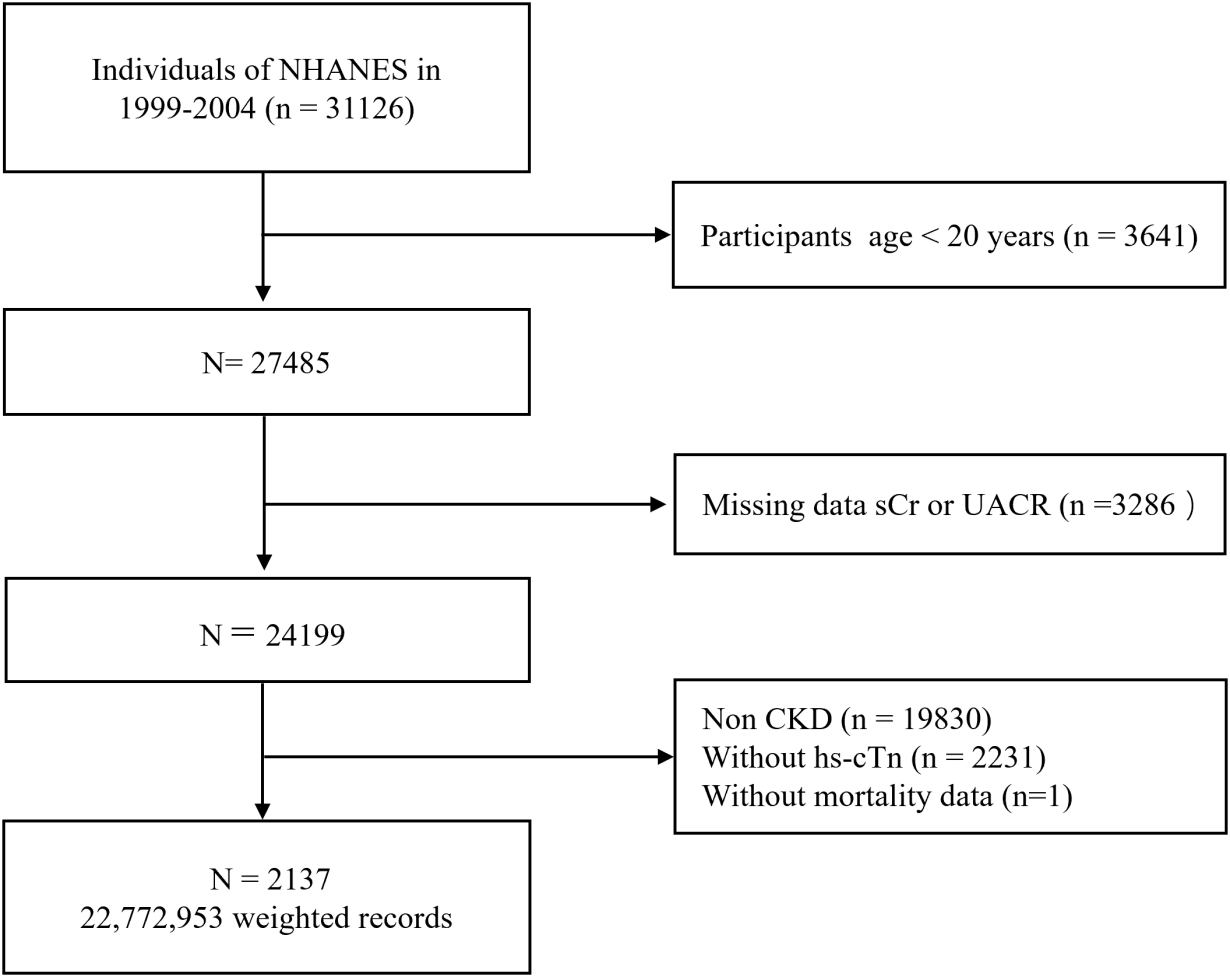
Flow chart of the participants in the study.

This study evaluates a biomarker phenotype rather than a clinical diagnosis of true pathophysiologic myocardial injury. The data for this retrospective study was derived from the National Health and Nutrition Examination Survey (NHANES) database, a program conducted by the National Center for Health Statistics (NCHS) of the Centers for Disease Control and Prevention (CDC). NHANES is a nationally representative survey that collects comprehensive information on the health and nutritional status of non-institutionalized civilian residents in the United States. The NHANES study protocol was approved by the National Center for Health Statistics Research Ethics Review Board, and all participants provided written informed consent [14]. We obtained NHANES data from 1999 to 2004. CKD was defined as an estimated glomerular filtration rate (eGFR) < 60 mL/min/1.73 m^2^ or urinary albumin-to-creatinine ratio (UACR) ≥ 30 mg/g, in accordance with the KDIGO criteria [15,16]. eGFR based on creatinine level was calculated using the Chronic Kidney Disease Epidemiology Collaboration (CKD-EPI) equation [17]. The CKD stages are typically defined as follows: Stage 1: Kidney damage with normal or increased GFR (eGFR ≥ 90 mL/min/1.73 m²); Stage 2: Mild reduction in GFR (eGFR 60–89 mL/min/1.73 m²); Stage 3: Moderate reduction in GFR (eGFR 30–59 mL/min/1.73 m²); Stage 4: Severe reduction in GFR (eGFR 15–29 mL/min/1.73 m²); Stage 5: Kidney failure (eGFR < 15 mL/min/1.73 m² or dialysis) [18]. Exclusion criteria included: (1) age < 20 years, (2) missing follow-up data, and (3) unavailable hs-cTn values. A total of 2,137 participants were included in the final analysis (Fig 1).

### Study sample

In September 2022, the NHANES program made available the hs-cTn dataset [19], which included hs-cTn measurements for participants from the 1999–2004 surveys who had serum sampled stored during that time and had authorized their use for their future research. The samples, preserved since the surveys, were analyzed between 2018 and 2020 at the University of Maryland School of Medicine in Baltimore, MD, USA. Four different assays were used for these measurements: hs-cTn T (Roche, using the Cobas e601), hs-cTn I (Abbott, using the ARCHITECT i2000SR), hs-cTn I (Siemens, using the Centaur XP), and hs-cTn I (Ortho, using the Vitros 3600).

### Assessment of covariates

The same variables were used in the analyses of both all-cause and cardiovascular mortality. Demographic and clinical characteristics included participant age, sex, race, body mass index (BMI), education level, smoking status, anemia, history of CVD, diabetes, hypertension, hyperlipidemia, total cholesterol (TC), triglycerides (TG), high-density lipoprotein cholesterol (HDL), low-density lipoprotein cholesterol (LDL), eGFR, urinary albumin-to-creatinine ratio (UACR), C-reactive protein (CRP), statin use, and use of angiotensin-converting enzyme inhibitor (ACEI)/angiotensin Ⅱ receptor blocker (ARB). Race groups were classified as Mexican American, other Hispanic, non-Hispanic white, non-Hispanic black, and others races. Educational levels were classified into three groups: “less than high school,” “high school or equivalent,” and “more than high school.” Smoking status was categorized into three groups: current smokers (individuals smoking daily or occasionally at the time of the survey), former smokers (those who had smoked at least 100 cigarettes in their lifetime but were not smoking at the time), and never smokers (fewer than 100 cigarettes smoked in their lifetime) [20]. Anemia was defined as hemoglobin level less than 13.0 g/dL for men and less than 12.0 g/dL for women (World Health Organization [WHO]). A history of CVD was identified based on self-reported physician diagnoses collected during a standardized medical interview. Participants were asked, “Has a doctor or other health expert ever told you that you have heart failure, chronic coronary heart disease, angina pectoris, myocardial infarction, or stroke?” Those responding “yes” to any of these conditions were classified as having CVD. According to the American Diabetes Association (ADA)’s diabetes diagnostic criteria, diabetes is defined by self-reported diagnosis, use of insulin or oral hypoglycemic medication, fasting blood glucose (FBG) ≥ 126 mg/dL, random blood glucose or 2h oral glucose tolerance test blood glucose ≥ 200 mg/dL, or glycosylated hemoglobin A1c (HbA1c) level 6.5% [21]. Hypertension was defined as a systolic blood pressure of 140 mmHg or higher, a diastolic blood pressure of 90 mmHg or greater, a self-reported diagnosis of hypertension by a physician, or the use of antihypertensive medications [22]. Hyperlipidemia was defined by the following criteria: LDL-C ≥ 130 mg/dL, TC ≥ 200 mg/dL, TG ≥ 150 mg/dL, or HDL-C ≤ 50 mg/dL in women and ≤ 40 mg/dL in men [23]. Additionally, individuals taking antihyperlipidemic medications were also classified as having hyperlipidemia [24].

### Statistical analysis

This study utilized advanced statistical methodologies to ensure that the results reflected the U.S non-institutionalized civilian population. Sample weights, as recommended by NHANES, were applied to address survey non-response, the intricacies of the sampling structure, and post-stratification, enhancing the accuracy of population estimates. Each NHANES survey cycle included a unique weighting variable, and for the analysis of data spanning 1999–2004, combined weights were computed according to NHANES protocols. Continuous variables, such as laboratory measurements and physical metrics, were summarized using medians along with interquartile ranges (IQR) to describe central values and variability. Categorical variables, including demographic and clinical characteristics, were presented as percentages to facilitate group comparisons. All analyses and estimates are based on weighted records unless otherwise specified, while unweighted counts are provided for transparency.

The definition and measurement of myocardial injury were central to this study. We defined myocardial injury biochemically as a single hs-cTn value exceeding the sex- and assay-specific 99th percentile upper reference limit. It is crucial to interpret this definition within the context of CKD, where an elevated hs-cTn may reflect a broad pathophysiological spectrum-including chronic myocardial strain, uremic cardiomyopathy, microvascular ischemia, and reduced renal clearance rather than exclusively indicating an acute ischemic event. Consequently, this criterion identifies a state of “troponin-defined myocardial injury” for the purpose of phenotyping and risk stratification. Operationally, this was identified using any of the four available hs-cTn assays with the following thresholds:

hs-cTnT: > 22 ng/L for men and >14 ng/L for women.
hs-cTn I (Abbott): > 35 ng/L for men and >17 ng/L for women.
hs-cTn I (Siemens): > 58 ng/L for men and >39.6 ng/L for women.
hs-cTn I (Ortho): > 12 ng/L for men and >9 ng/L for women [25].

No cross-assay standardization was attempted; each assay was interpreted using its manufacturer-specific, sex-stratified 99th percentile upper reference limit. The binary outcome of troponin-defined myocardial injury was defined as any assay exceeding its corresponding threshold, thereby aligning the clinical classification standard across platforms rather than assuming numerical equivalence of raw troponin values.

Mortality data, classified according to the International Classification of Diseases (ICD) codes, were derived from death certificates to ensure consistency in determining causes of death. Cardiovascular mortality included heart-related diseases (ICD codes I00–I09, I11, I13, and I20–I51) and cerebrovascular conditions (ICD codes I60–I69).

Pearson’s χ^2^ or Mann-Whitney U test was used to compare baseline characteristics as appropriate. The associations between myocardial injury and all-cause mortality were analyzed by Kaplan-Meier survival curves and Cox regression models. Kaplan-Meier curves were survey-weighted to incorporate the complex survey design (strata and primary sampling units). Survey-weighted Cox proportional hazards models were used for hazard ratio estimation, accounting for strata and primary sampling units as specified by NHANES analytic guidelines. Cumulative incidence at specific time points were estimated using unweighted data to provide observed estimates. Variables that were statistically significant in univariate analyses were incorporated into multivariate analyses. The full candidate variable list, with inclusion and exclusion rationale, is provided in S1 Table. In addition to statistically significant variables, we also included variables deemed potentially clinically relevant based on prior literature or clinical expertise, even if they did not reach statistical significance in univariate testing. Multicollinearity was formally assessing using the variance inflation factor (VIF), with VIF > 5 indicating high correlation and potential exclusion to ensure model stability (S2 Table). Model 1 was unadjusted. Model 2 was adjusted for age, sex, and race. Model 3 was adjusted for age, sex, race, stain use, and ACEI/ARB, which were forced into the model due to their clinical relevance. Additional covariates were selected from the remaining candidate list (S1 Table) using a stepwise forward selection procedure with entry and stay p-values of *P* < 0.10 and *P* < 0.05, respectively. The final set of covariates in Model 3 comprised the three forced-in variables and the stepwise-selected variables, which included:

- age
- sex
- race
- education level
- smoking status
- CVD
- diabetes
- hypertension
- hyperlipidemia
- anemia
- eGFR
- UACR
- CRP
- statin use
- ACEI/ARB use

We evaluated the proportional hazards assumption using the Schoenfeld residuals test, and no significant violations were detected (all *P* > 0.05), indicating a good model fit. The events-per-variable (EPV) ratio was 87.0 for all-cause mortality and 32.7 for cardiovascular mortality, both exceeding the recommended minimum threshold of EPV ≥ 10 [26]. The primary outcome of this analysis was the association between myocardial injury and long-term all-cause mortality in patients with CKD. A second focus was the association with long-term cardiovascular mortality. A formal test for effect modification was conducted to assess whether the association between myocardial injury and mortality differed by the specific hs-cTn assay used. This was performed by introducing an interaction term (assay type × myocardial injury status) into the primary Cox proportional hazards model (model 3). The significance of the interaction was evaluated using a likelihood ratio test, comparing the model with the interaction term to the nested model without it. To evaluate the robustness of our modeling approach, we performed sensitivity analyses using (1) a forced-entry Cox model with all candidate variables entered simultaneously, and (2) inverse probability of treatment weighting (IPTW) using propensity scores. To assess the isolated impact of myocardial injury, patients with pre-exisiting CVD were excluded in additional sensitivity analyses. All statistical analysis were conducted using R software (R-4.3.2). A *P*-value < 0.05 was considered statistically significant.

## Results

### Baseline characteristics

A total of 22,772,953 weighted records with CKD were included in the analysis, all of them were adults, with a minimum age of 20 year, and no missing vital status. The weighted records reflect the estimated population size; the actual participant count is 2,137 unweighted records. Those with troponin-defined myocardial injury, based on at least a single hs-cTn assay, accounted for 26.6% (6,067,451 records, 725 unweighted) of the cases. The distribution of the hs-cTn values in both arms of the study is available in Table 1. Table 2 presents the baseline characteristics of the cohort study participants. Patients with troponin-defined myocardial injury were older (77.0 vs 58.1, *P* < 0.001), more likely to be Non-Hispanic White race (75.8 vs 71.2%, *P* < 0.001) and Non-Hispanic Black (14.1 vs 9.2%, *P* < 0.001). They had higher rates of former smokers (41.5 vs 30.3%, *P* < 0.001) and current smokers (13.1 vs 2.2%, *P* < 0.001), and a greater prevalence of CVD (44.5 vs 15.4%, *P* < 0.001), hypertension (69.8 vs 47.3%, *P* < 0.001), diabetes (32.9 vs 23.2%, *P* < 0.001). Additionally, they exhibited higher uric acid levels (6.4 vs 5.8, *P* < 0.001), lower eGFR (50.1 vs 84.0, *P* < 0.001), and lower UACR (34.4 vs 41.3, *P* = 0.035) compared with those without troponin-defined myocardial injury.

**Table 1:** Median and interquartile range of troponin values grouped by the type of hs-cTn assay used to define myocardial injury.

hs-cTn assay^a^	Without myocardial Injury	Myocardial Injury
hs-cTn T. *n*	17,110,814	5,662,139
hs-cTn T. median (IQR), ng/L	8.7 (5.4-12.5)	27.5 (19.7-40.1)
hs-cTn I Abbott, *n*	21,751,377	1,021,576
hs-cTn I Abbott, median (IQR), ng/L	3.8 (2.2-7.0)	41.6 (22.9-63.4)
hs-cTn I Siemens, *n*	21,864,060	908,893
hs-cTn I Siemens, median (IQR), ng/L	5.4 (2.8-10.7)	87.6 (59.2-150.4)
hs-cTn I Ortho, *n*	21,269,001	1,503,952
hs-cTn I Ortho, median (IQR), ng/L	1.5 (0.6-3.3)	18.0 (13.4-30.8)

Abbreviations: hs-cTn, high-sensitivity cardiac troponin; IQR, Interquartile Range.

^a^All analyses and estimated are based on weighted records.

**Table 2:** Baseline characteristics of CKD patients.

Characteristics^a^	Without myocardial injury (*n* = 16705502; 73.4%)	Myocardial injury (*n* = 6067451; 26.6%)	*P*-value
Unweighted records, *n*	1412	725	
Age (years), median (IQR)	58.1 (42.0-72.0)	77.0 (68.0-84.0)	<0.001
Sex, female, *n* (%)	56.9	59.4	0.388
Race, *n* (%)			<0.001
Mexican American	6.6	3.3	
Other Hispanic	6.6	2.9	
Non-Hispanic White	71.2	75.8	
Non-Hispanic Black	9.2	14.1	
Other Race	16.4	3.8	
BMI, kg/m^2^, median (IQR)	28.2 (24.3-32.6)	27.7 (23.9-32.6)	0.224
Education levels, %			
Less than high school level	28.5	37.8	0.001
High school or equivalent	26.4	26.5	
Great than high school level	45.1	35.7	
Smoking status, %			
Ever	30.3	41.5	<0.001
Never	47.6	45.3	
Current	2.2	13.1	
History of CVD, %	15.4	44.5	<0.001
Hypertension, %	47.3	69.8	<0.001
Diabetes, %	23.2	32.9	<0.001
TC, mg/dL	201.0 (176.0-230.0)	200.0 (172.0-228.5)	0.466
TG, mg/dL	133.1 (91.0-199.0)	132.0 (90.0-191.0)	0.761
HDL, mg/dL	48.0 (39.0-59.0)	49.0 (40.5-61.0)	0.297
LDL, mg/dL	117.0 (96.0-141.0)	114.0 (93.0-137.0)	0.458
Uric acid, mg/dL	5.8 (4.6-6.8)	6.4 (5.2-7.6)	<0.001
eGFR, ml/min/1.73m^2^	84.0 (56.9-105.7)	50.1 (38.1-61.9)	<0.001
UACR, mg/g	41.3 (24.2-85.0)	34.4 (9.5-93.0)	0.035

CVD, cardiovascular disease; eGFR, estimated glomerular filtration rate; HDL, high density lipoprotein cholesterol; IQR, Interquartile Range; LDL, low density lipoprotein cholesterol; TC, total cholesterol; TG, triglyceride; UACR, urinary microalbumin creatinine ratio.

^a^All analyses and estimated are based on weighted records.

### Outcomes

**Fig 2:**
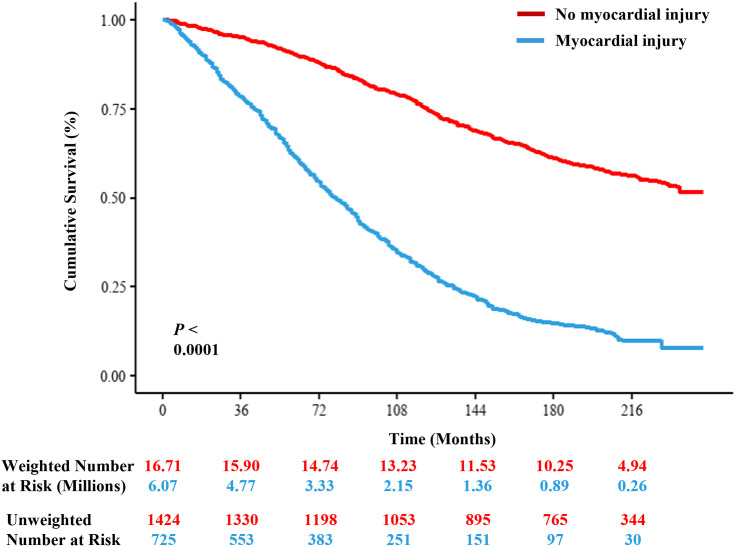
Kaplan-Meier survival curve for all-cause mortality in CKD patients using any hs-cTn assay to define myocardial injury.

The median follow-up duration was 11.6 years (IQR 5.8–16.8). Survival outcomes revealed that individuals with troponin-defined myocardial injury experienced significantly poorer survival probabilities compared with those without troponin-defined myocardial injury at various time points. At 1 year, survival probabilities were 94.0% for those with troponin-defined myocardial injury versus 98.4% for those without. This disparity widened over time, with survival probabilities declining to 62.0% versus 90.4% at 5 years, 30.1% versus 75.6% at 10 years, and 14.7% versus 61.2% at 15 years (Fig 2).

To address potential heterogeneity from pooling different hs-cTn assays, we formally tested for interaction between assay type and myocardial injury status on mortality. The likelihood ratio test for the interaction term was not statistically significant, indicating that the observed association between myocardial injury and mortality was consistent across the different assay types used in this study. Survival outcomes based on myocardial injury identified by different hs-cTn assays are summarized in Table 3 and shown in the corresponding Kaplan-Meier curves (S1–S4 Figs). After adjustment for baseline demographics and comorbid conditions using Cox proportional hazards models, troponin-defined myocardial injury was independently associated with increased risks of both all-cause and cardiovascular mortality. The adjusted hazard ratio (aHR) for all-cause mortality was 1.81 (95% CI 1.51–2.17, *P* < 0.001), and for cardiovascular mortality, it was 2.03 (95% CI 1.47–2.79, *P* < 0.001) compared with individuals without myocardial injury (Table 4 and S5 Fig). The associations varied substantially across different hs-cTn assay types. Among the assays, the hs-cTn I Ortho assay exhibited the strongest association with all-cause mortality (aHR 1.93, 95% CI 1.59–2.35, *P* < 0.001), while the hs-cTn I Abbott assay showed the strongest association with cardiovascular mortality (aHR 3.02, 95% CI 1.83–5.00, *P* < 0.001). These findings underscore the variability in prognostic implications depending on the specific assay used to define troponin-defined myocardial injury (Table 4).

**Table 3:** Unweighted cumulative survival of CKD patients grouped by type of hs-cTn assay used to define myocardial injury at 1, 5, 10, and 15 years.

hs-cTn assay/time point	Without myocardial injury	Myocardial injury
Any hs-cTn assay, *n* (unweighted)	16 705 502 (1412)	6 067 451 (725)
1 years, %	98.4	94.0
5 years, %	90.4	62.0
10 years, %	75.6	30.1
15 years, %	61.2	14.7
hs-cTn T, *n* (unweighted)	17,110,814 (1466)	5,662,139 (671)
1 years, %	98.5	93.5
5 years, %	90.4	60.1
10 years, %	75.5	27.4
15 years, %	60.9	12.4
hs-cTn I Abbott, *n* (unweighted)	21,751,377 (2014)	1,021,576 (123)
1 years, %	97.7	88.3
5 years, %	84.2	54.8
10 years, %	65.2	26.2
15 years, %	50.5	14.1
hs-cTn I Siemens, *n* (unweighted)	21,864,060 (2026)	908,893 (111)
1 years, %	97.6	89.8
5 years, %	83.7	62.3
10 years, %	64.7	33.2
15 years, %	50.1	17.9
hs-cTn I Ortho, *n* (unweighted)	21,269,001 (1945)	1,503,952 (192)
1 years, %	97.8	89.1
5 years, %	84.9	54.3
10 years, %	66.5	20.7
15 years, %	51.4	12.1

**Table 4:** Weighted hazard ratio for CKD patients with myocardial injury, defined by different types of hs-cTn assays (Cox regression).

Outcome	hs-cTn assay	Events (%)^a^	Model 1		Model 2		Model 3	
		Without myocardial injury *vs* myocardial injury	HR (95% CI)^b^	*P*-value	aHR (95% CI)^b^	*P*-value	aHR (95% CI)^b^	*P*-value
All-cause mortality	Any hs-cTn assay	733 (51.9) *vs* 659 (90.9)	4.09 (3.63-4.60)	< 0.001	2.03 (1.75-2.36)	< 0.001	1.81 (1.51-2.17)	< 0.001
	hs-cTn T	769 (52.5) *vs* 623 (92.9)	4.45 (3.95-5.02)	< 0.001	2.09 (1.79-2.45)	< 0.001	1.87 (1.55-2.25)	< 0.001
	hs-cTn I Abbott	1285 (63.8) *vs* 107 (87.0)	3.00 (2.40-3.73)	< 0.001	1.96 (1.53-2.50)	< 0.001	1.86 (1.40-2.49)	< 0.001
	hs-cTn I Siemens	1297 (64.0) *vs* 95 (85.6)	2.50 (1.97-3.16)	< 0.001	2.02 (1.58-2.57)	< 0.001	1.90 (1.42-2.52)	< 0.001
	hs-cTn I Ortho	1216 (62.5) *vs* 176 (91.7)	3.52 (2.93-4.24)	< 0.001	2.13 (1.72-2.64)	< 0.001	1.93 (1.59-2.35)	< 0.001
Cardiovascular mortality	Any hs-cTn assay	246 (17.4) *vs* 277 (38.2)	4.94 4.08-5.97)	< 0.001	1.80 (1.34-2.42)	< 0.001	2.03 (1.47-2.79)	< 0.001
	hs-cTn T	258 (17.6) *vs* 265 (39.4)	5.35 (4.41-6.48)	< 0.001	1.80 (1.31-2.48)	< 0.001	2.09 (1.48-2.95)	< 0.001
	hs-cTn I Abbott	467 (23.2) *vs* 56 (45.5)	4.79 (3.57-6.43)	< 0.001	3.36 (1.95-5.81)	< 0.001	3.02 (1.83-5.00)	< 0.001
	hs-cTn I Siemens	478 (23.6) *vs* 45 (40.5)	3.29 (2.34-4.61)	< 0.001	2.25 (1.24-4.09)	< 0.001	2.58 (1.57-4.23)	< 0.001
	hs-cTn I Ortho	437 (22.5) *vs* 86 (44.8)	5.18 4.00-6.72)	< 0.001	2.62 (1.61-4.26)	< 0.001	2.84 (1.86-4.33)	< 0.001

Multivariable analysis-all analyses and estimates are based on weighted records. Model 1 was unadjusted; Model 2 was adjusted for age, sex, race; Model 3 were adjusted for age, sex, race, education level, smoking status, CVD, diabetes, hypertension, anemia, dislipidemia, eGFR, UACR, CRP, statin drugs, ACEI/ARB drugs.

aHR, adjusted hazard ratio.

^a^unweighted, ^b^Reference group: CKD patients without myocardial injury.

In subgroup analyses stratified by CKD stage, troponin-defined myocardial injury was significantly associated with higher risks of all-cause and cardiovascular mortality in patients with CKD G1, G2 and G3. However, among patients with advanced CKD (G4-G5), this association was not statistically significant (Tables 5 and S3).

**Table 5:** Association of troponin-defined myocardial injury with mortality by CKD Stage.

Outcome	CKD stages	*n* (%)	aHR (95% CI)	*P*-value	*P* for interaction
All-cause mortality					0.496
	G1	619 (29.0)	2.62 (1.47-4.67)	< 0.001	
	G2	498 (23.2)	2.29 (1.64-3.19)	< 0.001	
	G3	914 (42.8)	1.54 (1.27-1.87)	< 0.001	
	G4-5	106 (5.0)	1.75 (0.50-6.11)	0.379	
Cardiovascular mortality					0.196
	G1	619 (29.0)	2.31 (1.91-5.88)	< 0.001	
	G2	498 (23.2)	1.97 (1.27-3.04)	< 0.001	
	G3	914 (42.8)	2.11 (1.43-3.10)	< 0.001	
	G4-5	106 (5.0)	6.20 (0.90-42.61)	0.064	

Multivariable analysis-all analyses and estimates are based on weighted records. Model were adjusted for age, sex, race, education level, smoking status, CVD, diabetes, hypertension, anemia, dislipidemia, eGFR, UACR, CRP, statin drugs, ACEI/ARB drugs.

aHR, adjusted hazard ratio.

### Sensitivity analysis excluding records with pre-existing cardiovascular disease

Two sensitivity approaches (forced entry and IPTW) yielded generally similar directional effects compared to the stepwise Model 3 (S4 Table), supporting the robustness of the primary findings to the variable selection strategy. The sensitivity analysis also examined a subgroup of participants with no prior CVD, comprising 17,494,332 survey responders (1,571 unweighted records). Of these, the majority (80.8%, 1,151 unweighted records) did not exhibit troponin-defined myocardial injury. The demographic, clinical, and laboratory characteristics of participants with and without troponin-defined myocardial injury was generally similar to those in the overall analysis. However, individuals with troponin-defined myocardial injury were characterized by a lower BMI and a lower prevalence of current smoking compared with their counterparts without myocardial injury (S5 Table).

**Fig 3:**
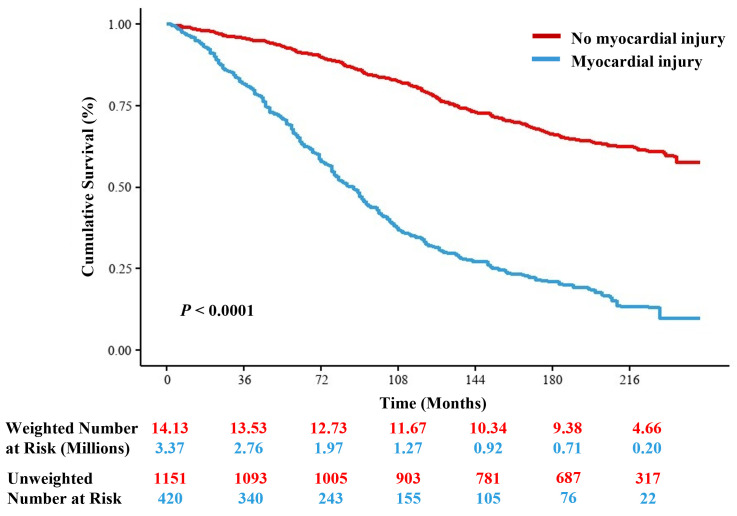
Kaplan-Meier survival curve for all-cause mortality in CKD patients with no prior cardiovascular disease using any hs-cTn assay to define myocardial injury.

Survival outcomes revealed significant disparities between participants with and without troponin-defined myocardial injury. At 1 year, individuals with troponin-defined myocardial injury had a survival rate of 96.1%, compared with 98.5% for those without. This survival gap widened over time, with survival probabilities declining to 66.0% vs 91.6% at 5 years, 33.2% vs 79.5% at 10 years, and 21.0% vs 66.3% at 15 years (Fig 3). Analyses stratified by the type of hs-cTn assay used to define myocardial injury revealed variations in survival outcomes, as detailed in S6 Table and visualized in S6–S9 Figs.

After adjusting for baseline characteristics using Cox regression models, troponin-defined myocardial injury was independently associated with significantly higher risks of both all-cause and cardiovascular mortality. The aHR for all-cause mortality was 1.86 (95% CI 1.56–2.21, *P* < 0.001), while the aHR for cardiovascular mortality was 2.44 (95% CI 1.83–3.24, *P* < 0.001) compared with participants without myocardial injury (S7 Table). Across all hs-cTn assays, individuals with troponin-defined myocardial injury consistently demonstrated elevated risks of mortality, reinforcing the prognostic value of detecting myocardial injury using hs-cTn assays.

## Discussion

This large, national, observational study examined long-term mortality in non-hospitalized patients with CKD. In this cohort study encompassing 22,772,953 weighted records of adults with CKD, we identified a significant association between troponin-defined myocardial injury (a biomarker phenotype) and adverse outcomes. We emphasize that our exposure is a biomarker phenotype and not a clinical diagnosis of acute or ongoing pathophysiologic myocardial injury. Our findings revealed that 26.6% of the study population had troponin-defined myocardial injury. Survival analysis demonstrated that individuals with troponin-defined myocardial injury had progressively worse survival probabilities compared with those without. After adjusting for baseline characteristics and comorbidities, troponin-defined myocardial injury showed a significant association with both all-cause and cardiovascular mortality in patients with CKD.

In this study, we defined myocardial injury using a single hs-cTn value exceeding the assay- and sex-specific 99th percentile. It is important to acknowledge the significant complexity in applying this biochemical definition to a CKD population. In patients with CKD, an elevated hs-cTn is a common finding that may not solely signify an acute ischemic event. It may reflect chronic pathological processes, such as sustained myocardial strain from volume and pressure overload, reduced renal clearance of troponin fragments, underlying structural heart disease, or subclinical uremic cardiomyopathy [27–29]. Consequently, our definition identifies a biochemical phenotype of myocardial involvement, encompassing a heterogeneous mix of acute and chronic etiologies. Notably, the strength of the associations varied depending on the specific hs-cTn assay used to define myocardial injury. The hs-cTn I Ortho assay demonstrated the highest risk for all-cause mortality, whereas the hs-cTn I Abbott assay was associated with the highest risk for cardiovascular mortality. This observation highlights the variability in risk prediction based on the assay employed. Furthermore, sensitivity analyses that excluded participants with pre-existing CVD confirmed the adverse impact of myocardial injury on survival outcomes. Collectively, these results underscore the prognostic significance of this troponin-defined biochemical phenotype in patients with CKD and emphasize that its interpretation and associated risk may depend on the specific hs-cTn assay used.

It is well-documented that CKD is closely linked to an increased risk of myocardial injury and cardiovascular events [30,31]. The mechanisms underlying this relationship are multifactorial: uremic toxins, oxidative stress, volume overload, renin-angiotensin-aldosterone-system (RAAS), sympathetic nervous system activation, CKD-mineral and bone disorder (CKD-MBD), malnutrition, and chronic inflammation can all contribute to subclinical myocardial stress or damage, as reflected by elevated hs-cTn levels [32]. Reduced eGFR is a key marker of kidney function that has been independently associated with increased cardiovascular risk and myocardial injury [33]. Elevated UACR reflects glomerular damage and serves as a marker of endothelial dysfunction, diffuse vascular damage, and systemic inflammation, which are pivotal in the development of CVD in CKD patients [34,35]. Unexpectedly, in our unadjusted analysis, CKD patients with troponin-defined myocardial injury had lower UACR levels compared to those without myocardial injury. However, after adjusting for demographic and clinical covariates (Model 3), the direction of this association reversed. The myocardial injury group showed higher log(UACR) (β = 0.14, 95% CI: −0.001 to 0.290, *P* = 0.051). This reversal indicates that the crude inverse association was confounded – particularly by lower eGFR and more frequent ACEI/ARB use in the myocardial injury group, both of which independently lower UACR – rather than reflecting a true inverse biological relationship. Notably, the conditions underlying such confounding-driven paradoxes, including competing risk structures, malnutrition-inflammation complexes, and survivorship biases, are precisely those described under the broader framework of reverse epidemiology in CKD [36]. This underscore the imperative for rigorous covariate adjustment when interpreting risk marker associations in this population.

In patients with CKD, chronic myocardial ischemia represents a major contributor to cardiovascular mortality, driven by a complex interplay of traditional and non-traditional risk factors that promote atherosclerotic CVD and acute coronary syndromes [37]. CKD specific alterations, including endothelial dysfunction, microvascular disease, chronic inflammation, and uremic toxicity, which increase plaque instability and heighten myocardial susceptibility to injury and adverse remodeling [38]. Consequently, the risk association between troponin-defined myocardial injury and cardiovascular mortality may reflect underlying microvascular dysfunction, accelerated fibrosis, inflammation, and plaque destabilization, ultimately culminating in cardiovascular death.

Our subgroup analyses suggest that the prognostic value of hs-cTn may vary across CKD stages. The attenuation of the troponin-mortality association in advanced CKD (G4-G5) warrants careful interpretation. While the wide confidence intervals (aHR 0.50–6.11 for all-cause mortality; aHR 0.90–42.61 for CVD mortality) are consistent with inadequate statistical power (n = 106, 5.0% of the cohort), several biological and methodological factors may also contribute. First, competing non-cardiovascular mortality in advanced CKD may dilute the association between a cardiovascular biomarker and all-cause mortality; notably, the CVD mortality point estimate (aHR = 6.20) was the largest across all stages, suggesting retained specificity for cardiovascular death. Second, the exceptionally high baseline mortality risk in G4-G5 may create a ceiling effect that limits the incremental prognostic value of any single biomarker. Third, the high prevalence of troponin elevation in advanced CKD reduces the biomarker’s discriminatory ability. The non-significant interaction tests were likely underpowered and cannot exclude a true modification effect. Future studies with larger advanced CKD cohorts and formal competing risk analyses are needed to disentangle these mechanisms. Of note, the IPTW sensitivity analysis yielded attenuated hazard ratios compared to the primary multivariable-adjusted model, particularly for cardiovascular mortality. This attenuation may reflect more effective confounding control by the propensity score weighting approach, and readers should interpret the primary estimates with this in mind. In sensitivity analysis excluding records with pre-existing CVD, we found that troponin-defined myocardial injury remained a significant predictor of adverse outcomes in CKD patients without known CVD. This highlights the profound impact of myocardial injury even in the absence of established cardiovascular conditions. Patients with troponin-defined myocardial injury had markedly lower survival probabilities at 1, 5, 10, and 15 years compared to those without myocardial injury, indicating a consistent trend of worse prognosis associated with troponin elevation. These findings align with previous studies that have demonstrated the independent prognostic value of elevated cardiac troponin levels in predicting mortality among patients without overt CVD [39,40].

This study has several important limitations that should be considered when interpreting the results. First, as many observational studies, the potential for residual confounding exists. Although we adjusted for numerous baseline characteristics and comorbidities, other unmeasured factors could have influenced the outcomes. Second, we deliberately employed an assay-specific threshold approach rather than cross-assay standardization, as no validated conversion method exists between hs-cTnT and hs-cTnI platforms, nor across different hs-cTnI assays. This strategy aligns the clinical classification standard across platforms but cannot fully eliminate the potential for differential classification of borderline cases. Third, despite our efforts to adjust for known cardiovascular disease and major confounders, we cannot rule out residual confounding from undiagnosed or subclinical cardiovascular disease, which may still influence the observed associations. Fourth, the data on CKD specific treatments received were not available, limiting our ability to adjust for these potentially significant factors. Fifth, the troponin measurements were performed at a single point in time, preventing analysis of trends and dynamic changes in troponin levels that could provide more nuanced insights into the worsening risk profile. Additionally, these measurements were conducted on stored frozen blood samples from many years ago, although recent evidence suggests that such assays remain reliable over long periods. Lastly, we acknowledge that the non-significant interaction test (*P* = 0.093) may reflect insufficient statistical power rather than true homogeneity of effects across assays. Interaction tests typically require substantially larger sample sizes than main effect analyses to detect the same magnitude of difference. The smallest assay subgroup had limited power to detect moderate heterogeneity. Therefore, the consistency of the overall association should not be interpreted as evidence that the prognostic value is identical across all assays, and future studies with larger assay-specific subgroups are warranted. While we demonstrate a strong independent association, we cannot infer a causal relationship between troponin-defined myocardial injury and mortality. Our findings identify hs-cTn as a powerful risk stratification marker, not a modifiable risk factor. These limitations highlight the need for further research with more detailed and longitudinal data to validate and extend our findings.

## Conclusion

In conclusion, troponin-defined myocardial injury, identified by elevated hs-cTn, was independently associated with adverse long-term outcomes in patients with CKD. This study supports the role of hs-cTn elevation as a risk stratification marker in the CKD population. However, our findings do not justify the immediate implementation of routine hs-cTn screening in asymptomatic patients with CKD. Whether hs-cTn-guided management strategies can improve clinical outcomes remains unknown and requires evaluation in future prospective interventional studies.

## Supporting information

S1 Table Candidate variables were considered for inclusion in the multivariable models. BMI, body mass index; CRP, C-reative protein; CVD, cardiovascular disease; eGFR, estimated glomerular filtration rate; HDL, high density lipoprotein cholesterol; LDL, low density lipoprotein cholesterol; TC, total cholesterol; TG, triglyceride; UACR, urinary microalbumin creatinine ratio.
(DOCX)

S2 Table Variance inflation factor of the adjusted covariates. BMI, body mass index; CRP, C-reative protein; CVD, cardiovascular disease; eGFR, estimated glomerular filtration rate; UACR, urinary microalbumin creatinine ratio.
(DOCX)

S3 Table Troponin-defined myocardial injury prevalence and mortality by CKD stages. (DOCX)

S4 Table Comparison of hazard ratios for the association between troponin-defined myocardial injury and mortality using alternative modeling strategies versus stepwise multivariable model. Model were adjusted for age, sex, race, education level, smoking status, CVD, diabetes, hypertension, anemia, dislipidemia, eGFR, UACR, CRP, statin drugs, ACEI/ARB drugs. aHR, adjusted hazard ratio. ^a^ Reference group: CKD patients without myocardial injury.
(DOCX)

S5 Table Baseline records characteristics of CKD patients with no prior cardiovascular disease. BMI, body mass index; TC, total cholesterol; TG, triglyceride; HDL, high density lipoprotein cholesterol; LDL, low density lipoprotein cholesterol; eGFR, estimated glomerular filtration rate; UACR, urinary microalbumin creatinine ratio. ^a^ All analyses and estimated are based on weighted records.
(DOCX)

S6 Table Unweighted cumulative survival of CKD patients with no know cardiovascular disease stratified by type of hs-cTn assay used to defined myocardial injury at 1, 5, 10, and 15 years. (DOCX)

S7 Table Weighted hazard ratio myocardial injury in CKD patients without cardiovascular disease, defined by different types of hs-cTn assay used to define myocardial injury (Cox regression). (DOCX)

S1 Fig Kaplan-Meier survival curve for all-cause mortality in CKD patients using hs-cTn T assay to define myocardial injury. (TIF)

S2 Fig Kaplan-Meier survival curve for all-cause mortality in CKD patients using Abbott hs-cTn I assay to define myocardial injury. (TIF)

S3 Fig Kaplan-Meier survival curve for all-cause mortality in CKD patients using Siements hs-cTn I assay to define myocardial injury. (TIF)

S4 Fig Kaplan-Meier survival curve for all-cause mortality in CKD patients using Ortho hs-cTn I assay to define myocardial injury. (TIF)

S5 Fig Assay-stratified associations between troponin-defined myocardial injury and mortality in patients with CKD. Adjusted hazard ratios are shown for all-cause mortality and cardiovascular mortality stratified by hs-cTn assay platform. Diamonds represent pooled estimates across all assays; squares represent individual assay estimates with 95% confidence intervals. Model was adjusted for age, sex, race, education, smoking, cardiovascular disease, diabetes, hypertension, anemia, dyslipidemia, eGFR, UACR, CRP, statin use, and ACEI/ARB use.
(TIF)

S6 Fig Kaplan-Meier survival curve for all-cause mortality in CKD patients with no known cardiovascular disease using hs-cTn T assay to define myocardial injury. (TIF)

S7 Fig Kaplan-Meier survival curve for all-cause mortality in CKD patients with no known cardiovascular disease using Abbott hs-cTn I assay to define myocardial injury. (TIF)

S8 Fig Kaplan-Meier survival curve for all-cause mortality in CKD patients with no known cardiovascular disease using Siements hs-cTn I assay to define myocardial injury. (TIF)

S9 Fig Kaplan-Meier survival curve for all-cause mortality in CKD patients with no known cardiovascular disease using Ortho hs-cTn I assay to define myocardial injury. (TIF)
