# Supplementary material for: Association of troponin-defined myocardial injury with adverse long-term survival among patients with chronic kidney disease
Source: PLoS One. 2026 Jul 30;21(7):e0354873. doi: 10.1371/journal.pone.0354873 (PMC13422838; doi:10.1371/journal.pone.0354873)
Supplement: S1 Table — BMI, body mass index; CRP, C-reative protein; CVD, cardiovascular disease; eGFR, estimated glomerular filtration rate; HDL, high density lipoprotein cholesterol; LDL, low density lipoprotein cholesterol; TC, total cholesterol; TG, triglyceride; UACR, urinary microalbumin creatinine ratio. (DOCX) [file pone.0354873.s001.docx]

|  | Variable | Type | Rationale |
| --- | --- | --- | --- |
| 1 | Age | Continuous | Demographic; forced entry |
| 2 | Sex | Categorical | Demographic; forced entry |
| 3 | Race | Categorical | Demographic; forced entry |
| 4 | BMI | Continuous | Clinical relevance |
| 5 | Education level | Categorical | Socioeconomic confounder |
| 6 | Smoking status | Categorical | Major cardiovascular risk factor |
| 7 | Anemia | Categorical | Clinical relevance in CKD |
| 8 | CVD | Categorical | Major confounder |
| 9 | Diabetes | Categorical | Major cardiovascular risk factor |
| 10 | Hypertension | Categorical | Major cardiovascular risk factor |
| 11 | Hyperlipidemia | Categorical | Cardiovascular risk factor |
| 12 | TC | Continuous | Lipid metabolism |
| 13 | TG | Continuous | Lipid metabolism |
| 14 | HDL | Continuous | Lipid metabolism |
| 15 | LDL | Continuous | Lipid metabolism |
| 16 | eGFR | Continuous | Kidney function |
| 17 | UACR | Continuous | Kidney damage marker |
| 18 | CRP | Continuous | Inflammation |
| 19 | Statin use | Categorical | Clinical relevance; forced entry |
| 20 | ACEI/ARB use | Categorical | Clinical relevance; forced entry |
| 21 | Myocardial injury (exposure) | Categorical | Primary exposure variable |

**Supplemental Table 1.** Candidate variables were considered for inclusion in the multivariable models

BMI, body mass index; CRP, C-reative protein; CVD, cardiovascular disease; eGFR, estimated glomerular filtration rate; HDL, high density lipoprotein cholesterol; LDL, low density lipoprotein cholesterol; TC, total cholesterol; TG, triglyceride; UACR, urinary microalbumin creatinine ratio.
