# Supplementary material for: Association of troponin-defined myocardial injury with adverse long-term survival among patients with chronic kidney disease
Source: PLoS One. 2026 Jul 30;21(7):e0354873. doi: 10.1371/journal.pone.0354873 (PMC13422838; doi:10.1371/journal.pone.0354873)
Supplement: S2 Table — BMI, body mass index; CRP, C-reative protein; CVD, cardiovascular disease; eGFR, estimated glomerular filtration rate; UACR, urinary microalbumin creatinine ratio. (DOCX) [file pone.0354873.s002.docx]

**Supplemental Table 2.** Variance inflation factor of the adjusted covariates

|  | VIF |
| --- | --- |
| Myocardial injury | 1.31 |
| Age | 2.01 |
| Race | 1.07 |
| Sex | 1.10 |
| BMI | 1.14 |
| Education level | 1.08 |
| Smoking status | 1.09 |
| CVD | 1.18 |
| Diabetes | 1.20 |
| Hypertension | 1.21 |
| Anemia | 1.08 |
| dislipidemia | 1.13 |
| eGFR | 1.99 |
| UACR | 1.07 |
| CRP | 1.02 |
| Statin drug | 1.08 |
| ACEI/ARB drug | 1.05 |

BMI, body mass index; CRP, C-reative protein; CVD, cardiovascular disease; eGFR, estimated glomerular filtration rate; UACR, urinary microalbumin creatinine ratio.
