# Supplementary material for: Association of troponin-defined myocardial injury with adverse long-term survival among patients with chronic kidney disease
Source: PLoS One. 2026 Jul 30;21(7):e0354873. doi: 10.1371/journal.pone.0354873 (PMC13422838; doi:10.1371/journal.pone.0354873)
Supplement: S3 Table — (DOCX) [file pone.0354873.s003.docx]

**Supplemental Table 3.** Troponin-defined myocardial injury prevalence and mortality by CKD stages

| CKD stages | *n* | Myocardial injury, *n* (%) | All-cause mortality, *n* (%) | | Cardiovascular-mortality, *n* (%) | |
| --- | --- | --- | --- | --- | --- | --- |
|  |  |  | Without myocardial injury | Myocardial injury | Without myocardial injury | Myocardial injury |
| G1 | 619 | 61 (9.9) | 154 (27.6) | 41 (67.2) | 51 (9.1) | 13 (21.3) |
| G2 | 498 | 146 (29.3) | 227 (64.5) | 139 (95.2) | 89 (25.3) | 58 (39.7) |
| G3 | 914 | 423 (46.3) | 343 (69.9) | 392 (92.7) | 103 (21.0) | 167 (39.5) |
| G4-5 | 106 | 95 (89.6) | 9 (81.8) | 90 (94.7) | 3 (27.3) | 39 (41.1) |
