# Supplementary material for: Association of troponin-defined myocardial injury with adverse long-term survival among patients with chronic kidney disease
Source: PLoS One. 2026 Jul 30;21(7):e0354873. doi: 10.1371/journal.pone.0354873 (PMC13422838; doi:10.1371/journal.pone.0354873)
Supplement: S4 Table — Model were adjusted for age, sex, race, education level, smoking status, CVD, diabetes, hypertension, anemia, dislipidemia, eGFR, UACR, CRP, statin drugs, ACEI/ARB drugs. aHR, adjusted hazard ratio. a Reference group: CKD patients without myocardial injury. (DOCX) [file pone.0354873.s004.docx]

**Supplemental Table 4.** Comparison of hazard ratios for the association between troponin-defined myocardial injury and mortality using alternative modeling strategies versus stepwise multivariable Model.

| Outcome | hs-cTn assay | Forced-entry | | IPTW-weighted | |
| --- | --- | --- | --- | --- | --- |
|  |  | aHR (95% CI)^a^ | *P*-value | aHR (95% CI)^a^ | *P*-value |
| All-cause mortality | Any hs-cTn assay | 1.79 (1.54-2.09) | < 0.001 | 1.50 (1.09-2.06) | 0.012 |
|  | hs-cTn T | 1.85 (1.58-2.17) | < 0.001 | 1.70 (1.13-2.58) | 0.011 |
|  | hs-cTn I Abbott | 1.68 (1.24-2.27) | < 0.001 | 1.77 (1.22-2.56) | 0.002 |
|  | hs-cTn I Siemens | 1.90 (1.42-2.54) | < 0.001 | 1.37 (1.00-1.93) | 0.042 |
|  | hs-cTn I Ortho | 1.80 (1.46-2.23) | < 0.001 | 1.23 (1.05-2.49) | 0.048 |
| Cardiovascular mortality | Any hs-cTn assay | 2.13 (1.64-2.76) | < 0.001 | 1.60 (1.03-2.50) | 0.038 |
|  | hs-cTn T | 2.16 (1.62-2.89) | < 0.001 | 1.49 (1.01-2.46) | 0.023 |
|  | hs-cTn I Abbott | 2.60 (1.73-3.89) | < 0.001 | 2.11 (1.22-3.65) | 0.007 |
|  | hs-cTn I Siemens | 2.45 (1.53-3.93) | < 0.001 | 1.77 (1.09-2.86) | 0.021 |
|  | hs-cTn I Ortho | 2.58 (1.87-3.59) | < 0.001 | 1.21 (1.04-1.64) | 0.046 |

Model were adjusted for age, sex, race, education level, smoking status, CVD, diabetes, hypertension, anemia, dislipidemia, eGFR, UACR, CRP, statin drugs, ACEI/ARB drugs.

aHR, adjusted hazard ratio.

^a^ Reference group: CKD patients without myocardial injury.
