# Supplementary material for: Association of troponin-defined myocardial injury with adverse long-term survival among patients with chronic kidney disease
Source: PLoS One. 2026 Jul 30;21(7):e0354873. doi: 10.1371/journal.pone.0354873 (PMC13422838; doi:10.1371/journal.pone.0354873)
Supplement: S5 Table — BMI, body mass index; TC, total cholesterol; TG, triglyceride; HDL, high density lipoprotein cholesterol; LDL, low density lipoprotein cholesterol; eGFR, estimated glomerular filtration rate; UACR, urinary microalbumin creatinine ratio. a All analyses and estimated are based on weighted records. (DOCX) [file pone.0354873.s005.docx]

**Supplemental Table 5.** Baseline records characteristics of CKD patients with no prior cardiovascular disease

| Characteristics^a^ | Without myocardial injury (*n* = 14128079; 80.8%) | Myocardial injury (*n* = 3366253; 19.2% ) | *P*-value |
| --- | --- | --- | --- |
| Unweighted records, *n* | 1151 | 420 |  |
| Age (years), median (IQR) | 55.0 (40.0-70.0) | 77.0 (67.0-84.0) | <0.001 |
| Sex, female, % | 58.0 | 60.6 | 0.448 |
| Race (%) |  |  | <0.001 |
| Mexican American | 7.2 | 3.9 |  |
| Other Hispanic | 7.4 | 4.4 |  |
| Non-Hispanic White | 68.9 | 74.8 |  |
| Non-Hispanic Black | 9.3 | 4.9 |  |
| Other Race | 7.2 | 2.0 |  |
| BMI, kg/m^2^, median (IQR) | 28.2 (24.3-32.8) | 26.3 (22.7-31.9) | 0.002 |
| Education levels, % |  |  | 0.016 |
| Less than high school level | 27.6 | 35.0 |  |
| High school or equivalent | 27.1 | 29.3 |  |
| Great than high school level | 45.3 | 35.7 |  |
| Smoking status, % |  |  | 0.004 |
| Ever | 28.4 | 37.1 |  |
| Never | 48.6 | 48.3 |  |
| Current | 23.1 | 14.6 |  |
| Hypertension, % | 42.8 | 65.5 | <0.001 |
| Diabetes, % | 21.9 | 25.5 | 0.22 |
| TC, mg/dL | 202.0 (177.0-231.0) | 206.0 (176.0-234.0) | 0.505 |
| TG, mg/dL | 128.0 (90.0-194.0) | 117.0 (80.0-168.4) | 0.019 |
| HDL, mg/dL | 48.0 (39.0- 59.0) | 51.0 (41.0-68.0) | 0.006 |
| LDL, mg/dL | 120.0 (97.0-142.0) | 116.0 (95.4-138.8) | 0.498 |
| Uric acid, mg/dL | 5.6 (4.6-6.7) | 6.0 (4.8-7.1) | 0.001 |
| eGFR, ml/min/1.73m^2^ | 88.6 (58.5-108.8) | 53.0 (41.5-70.9) | <0.001 |
| UACR, mg/g | 42.2 (30.5-83.5) | 35.0 (10.7-87.9) | 0.049 |

BMI, body mass index; TC, total cholesterol; TG, triglyceride; HDL, high density lipoprotein cholesterol; LDL, low density lipoprotein cholesterol; eGFR, estimated glomerular filtration rate; UACR, urinary microalbumin creatinine ratio.

^a^ All analyses and estimated are based on weighted records.
