# Supplementary material for: Association of troponin-defined myocardial injury with adverse long-term survival among patients with chronic kidney disease
Source: PLoS One. 2026 Jul 30;21(7):e0354873. doi: 10.1371/journal.pone.0354873 (PMC13422838; doi:10.1371/journal.pone.0354873)
Supplement: S6 Table — (DOCX) [file pone.0354873.s006.docx]

**Supplemental Table 6.** Unweighted cumulative survival of CKD patients with no know cardiovascular disease stratified by type of hs-cTn assay used to defined myocardial injury at 1, 5, 10, and 15 years

| hs-cTn assay/time point | Without myocardial injury | Myocardial injury |
| --- | --- | --- |
| Any hs-cTn assay, *n* (unweighted) | 14,128,079 (1151) | 3,366,253 (420) |
| 1 years, % | 98.5 | 96.1 |
| 5 years, % | 91.6 | 66.0 |
| 10 years, % | 79.5 | 33.2 |
| 15 years, % | 66.3 | 21.0 |
| hs-cTn T, *n* (unweighted) | 14,432,736 (1186) | 3,061,596 (385) |
| 1 years, % | 98.6 | 95.7 |
| 5 years, % | 91.5 | 63.8 |
| 10 years, % | 79.3 | 29.5 |
| 15 years, % | 66.1 | 17.4 |
| hs-cTn I Abbott, *n* (unweighted) | 16,973,692 (1503) | 520,640 (68) |
| 1 years, % | 98.2 | 94.2 |
| 5 years, % | 87.5 | 58.8 |
| 10 years, % | 71.8 | 29.7 |
| 15 years, % | 58.8 | 18.8 |
| hs-cTn I Siements, *n* (unweighted) | 16,981,553 (1507) | 512,779 (64) |
| 1 years, % | 98.2 | 94.8 |
| 5 years, % | 87.3 | 66.9 |
| 10 years, % | 71.7 | 34.6 |
| 15 years, % | 58.6 | 21.8 |
| hs-cTn I Ortho, *n* (unweighted) | 16,798,080 (1481) | 696,252 (90) |
| 1 years, % | 98.3 | 92.4 |
| 5 years, % | 87.7 | 61.7 |
| 10 years, % | 72.3 | 29.9 |
| 15 years, % | 59.1 | 20.3 |
