# Supplementary material for: Association of troponin-defined myocardial injury with adverse long-term survival among patients with chronic kidney disease
Source: PLoS One. 2026 Jul 30;21(7):e0354873. doi: 10.1371/journal.pone.0354873 (PMC13422838; doi:10.1371/journal.pone.0354873)
Supplement: S7 Table — (DOCX) [file pone.0354873.s007.docx]

**Supplemental Table 7.** Weighted hazard ratio myocardial injury in CKD patients without cardiovascular disease, defined by different types of hs-cTn assay used to define myocardial injury (Cox regression)

| Outcome | hs-cTn assay | Events (%)^a^ | Model 1 | | Model 2 | | Model 3 | |
| --- | --- | --- | --- | --- | --- | --- | --- | --- |
|  |  | Without myocardial injury *vs* myocardial injury | HR (95% CI)^b^ | *P*-value | aHR (95% CI)^b^ | *P*-value | aHR (95% CI)^b^ | *P*-value |
| All-cause mortality | Any hs-cTn assay | 528 (37.7) *vs* 369 (84.7) | 4.25 (3.64-4.96) | < 0.001 | 1.85 (1.57-2.19) | < 0.001 | 1.86 (1.56-2.21) | < 0.001 |
|  | hs-cTn T | 547 (37.9) *vs* 350 (88.5) | 4.79 (4.10-5.61) | < 0.001 | 1.90 (1.61-2.25) | < 0.001 | 1.90 (1.59-2.26) | < 0.001 |
|  | hs-cTn I Abbott | 841 (45.6) *vs* 56 (84.6) | 3.41 (2.49-4.69) | < 0.001 | 1.91 (1.39-2.64) | < 0.001 | 1.83 (1.31-2.55) | < 0.001 |
|  | hs-cTn I Siemens | 846 (45.7) *vs* 51 (80.6) | 2.88 (1.97-3.16) | < 0.001 | 1.91 (1.37-265) | < 0.001 | 1.94 (1.39-2.71) | < 0.001 |
|  | hs-cTn I Ortho | 819 (45.2) *vs* 78 (84.5) | 3.44 (2.60-4.54) | < 0.001 | 1.75 (1.32-2.32) | < 0.001 | 1.64 (1.22-2.20) | < 0.001 |
| Cardiovascular mortality | Any hs-cTn assay | 163(12.0) *vs* 151 (34.9) | 5.54 4.30-7.14) | < 0.001 | 2.46 (1.87-3.23) | < 0.001 | 2.44 (1.83-3.24) | < 0.001 |
|  | hs-cTn T | 170 (12.1) *vs* 144 (36.4) | 6.20 (4.79-8.02) | < 0.001 | 2.51 (1.90-3.31) | < 0.001 | 2.46 (1.84-3.29) | < 0.001 |
|  | hs-cTn I Abbott | 288 (15.5) *vs* 26 (42.8) | 5.01 (3.19-7.88) | < 0.001 | 2.77 (1.75-4.40) | < 0.001 | 2.73 (1.69-4.40) | < 0.001 |
|  | hs-cTn I Siemens | 293 (15.9) *vs* 21 (33.2) | 3.40 (2.03-5.68) | < 0.001 | 2.18 (1.30-3.65) | < 0.001 | 2.58 (1.57-4.25) | 0.010 |
|  | hs-cTn I Ortho | 277 (15.3) *vs* 37 (42.7) | 5.10 (3.42-7.61) | < 0.001 | 2.56 (1.70-3.86) | < 0.001 | 2.49 (1.62-3.84) | < 0.001 |

Multivariable analysis-all analyses and estimates are based on weighted records. Model 1 was unadjusted; Model 2 was adjusted for age, sex, race; Model 3 were adjusted for age, sex, race, education level, smoking status, diabetes, hypertension, anemia, dislipidemia, eGFR, UACR, CRP, statin drugs, ACEI/ARB drugs.

aHR, adjusted hazard ratio.

^a^ unweighted, ^b^Reference group: CKD patients without myocardial injury
