# Supplementary figures and images for: Association of troponin-defined myocardial injury with adverse long-term survival among patients with chronic kidney disease
Source: PLoS One. 2026 Jul 30;21(7):e0354873. doi: 10.1371/journal.pone.0354873 (PMC13422838; doi:10.1371/journal.pone.0354873)

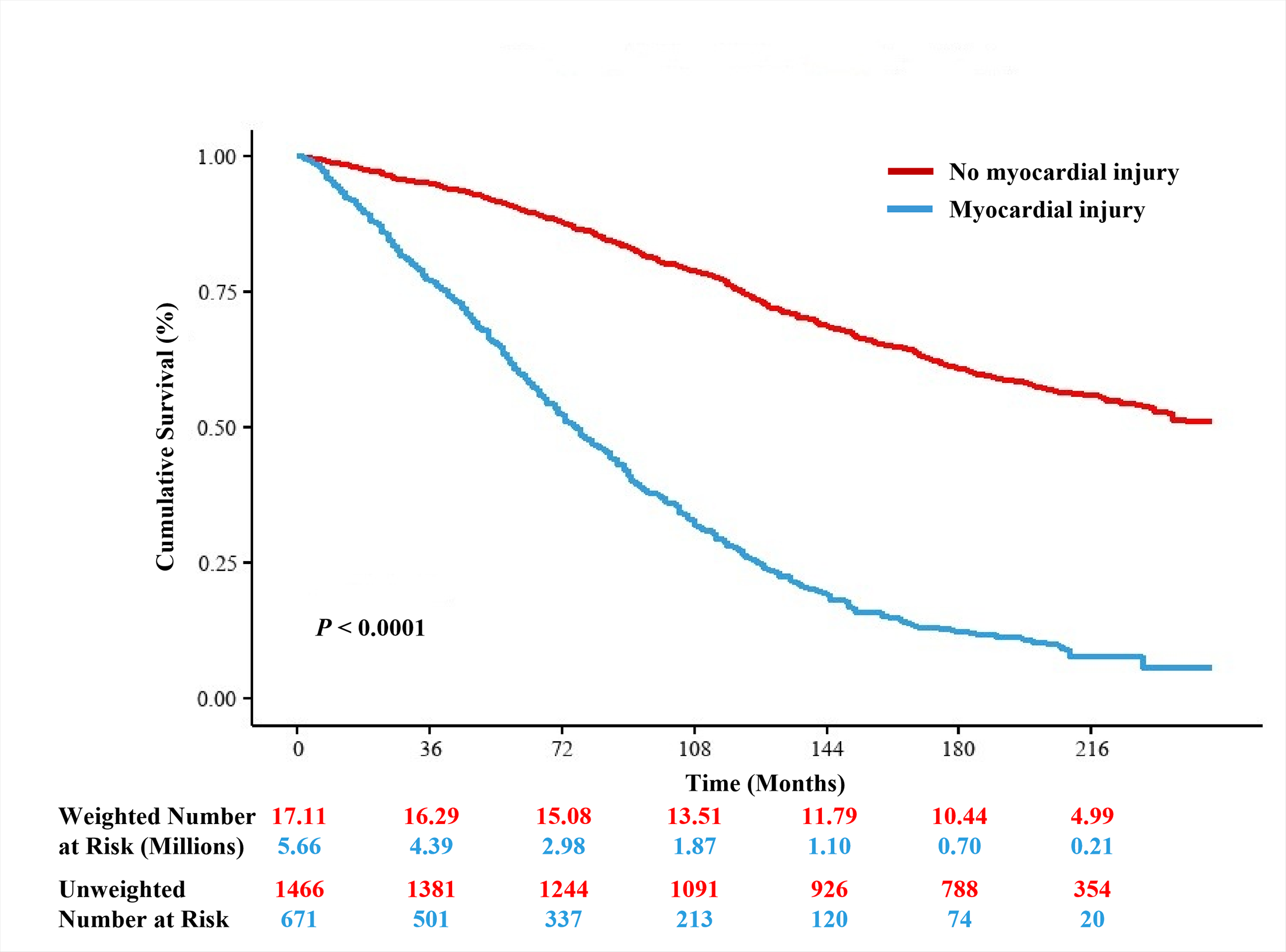

Supplement: S1 Fig — (TIF) [file pone.0354873.s008.tif]

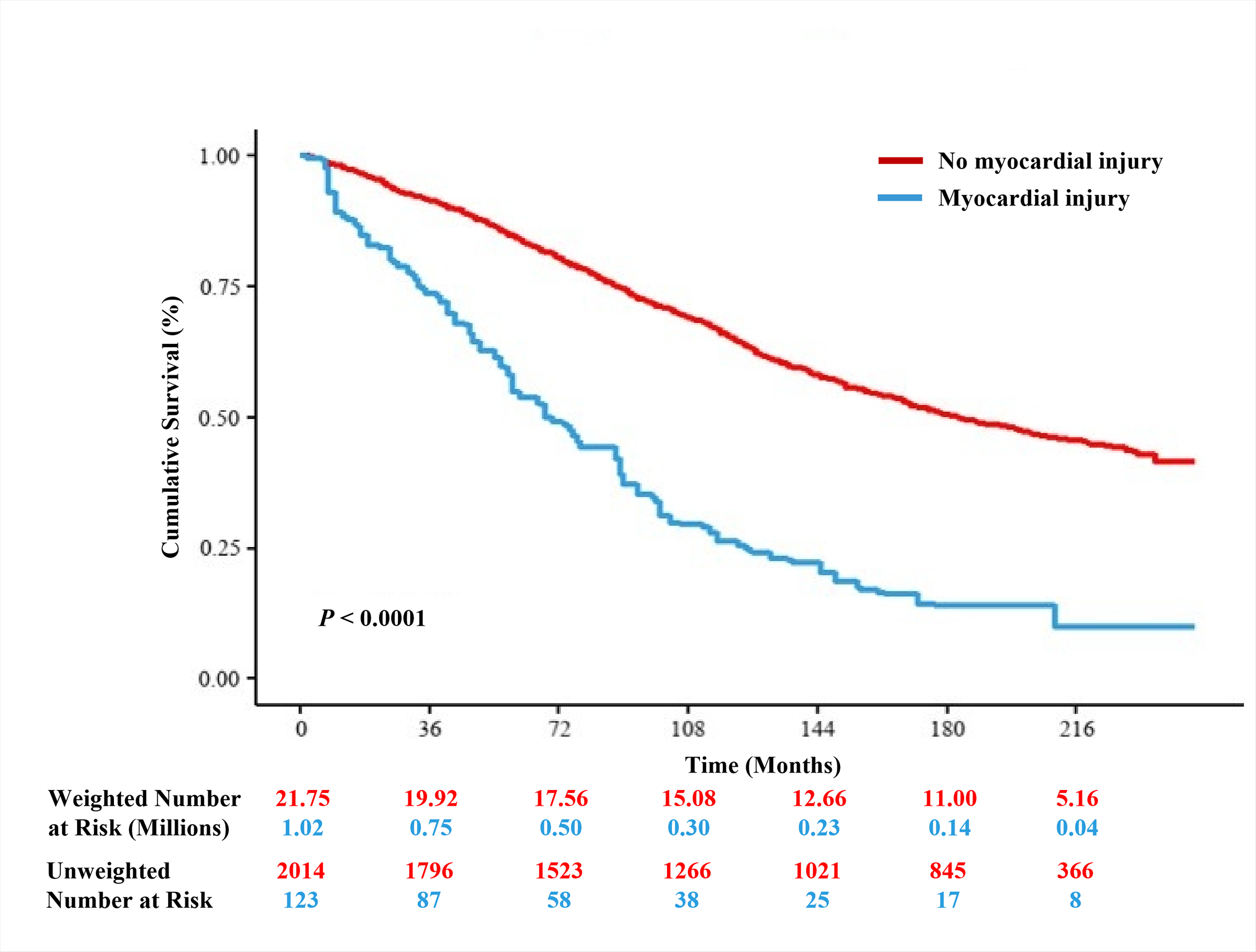

Supplement: S2 Fig — (TIF) [file pone.0354873.s009.tif]

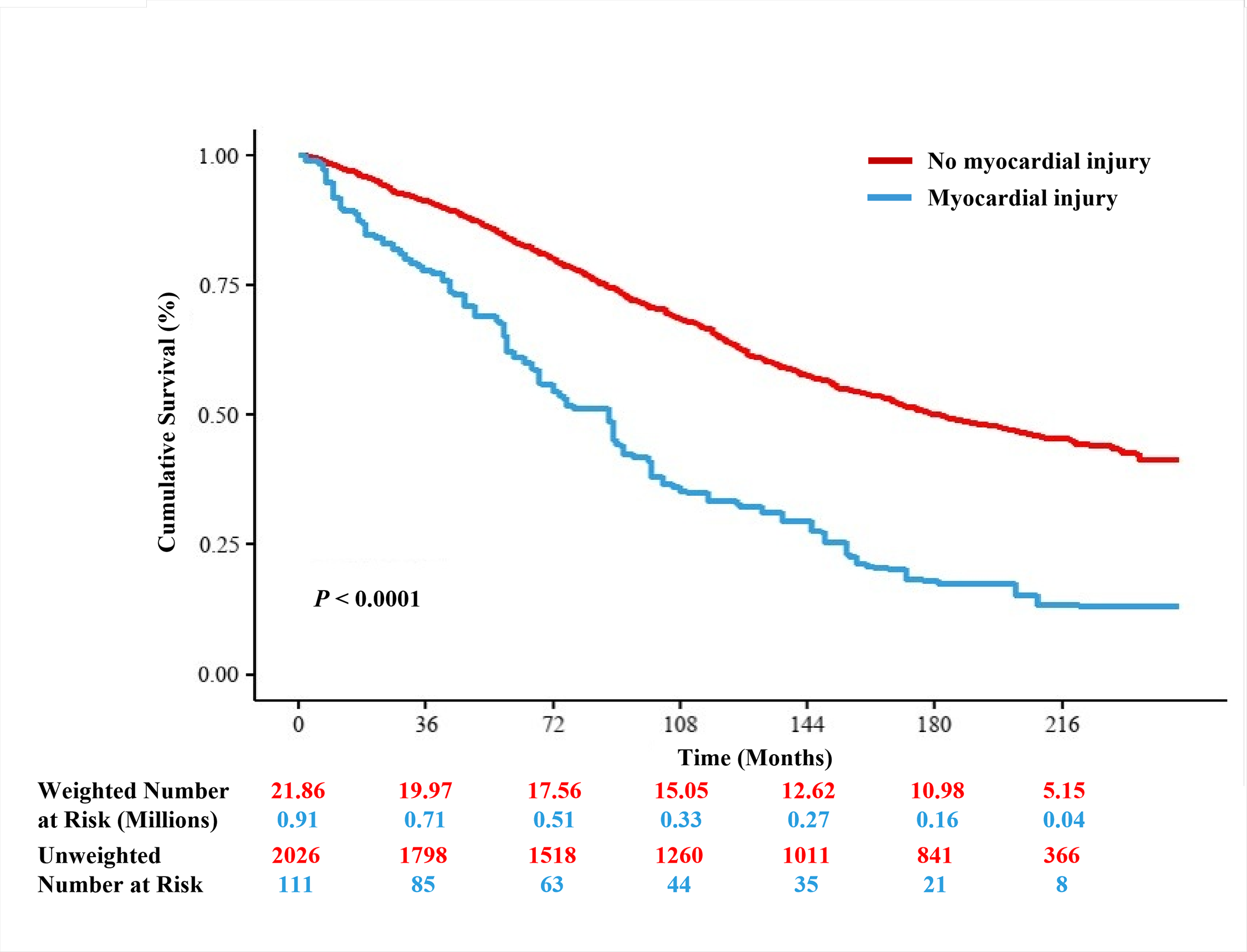

Supplement: S3 Fig — (TIF) [file pone.0354873.s010.tif]

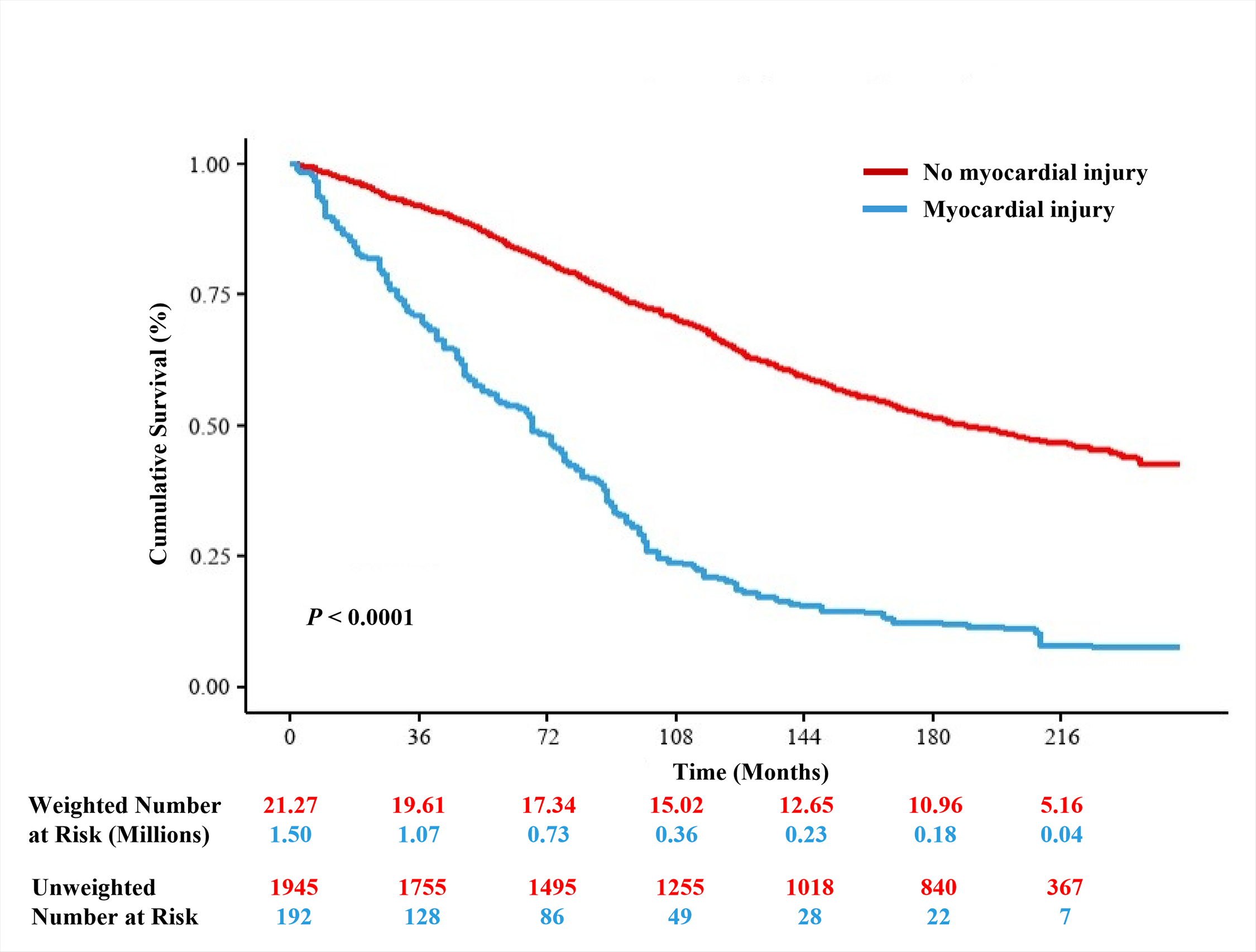

Supplement: S4 Fig — (TIF) [file pone.0354873.s011.tif]

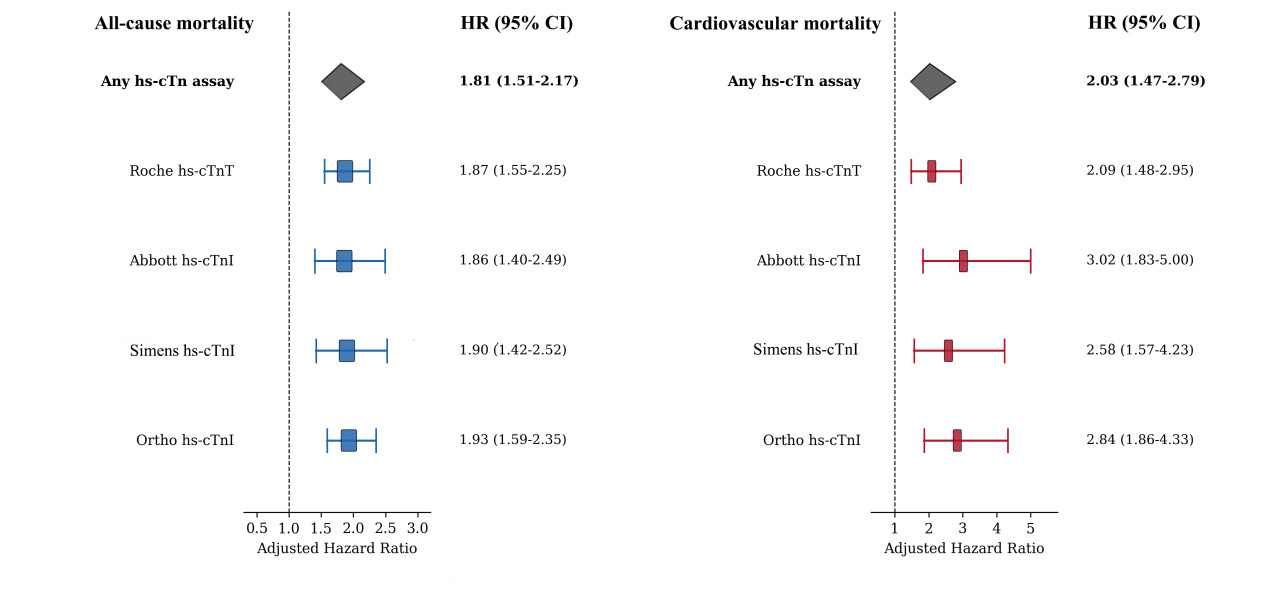

Supplement: S5 Fig — Adjusted hazard ratios are shown for all-cause mortality and cardiovascular mortality stratified by hs-cTn assay platform. Diamonds represent pooled estimates across all assays; squares represent individual assay estimates with 95% confidence intervals. Model was adjusted for age, sex, race, education, smoking, cardiovascular disease, diabetes, hypertension, anemia, dyslipidemia, eGFR, UACR, CRP, statin use, and ACEI/ARB use. (TIF) [file pone.0354873.s012.tif]

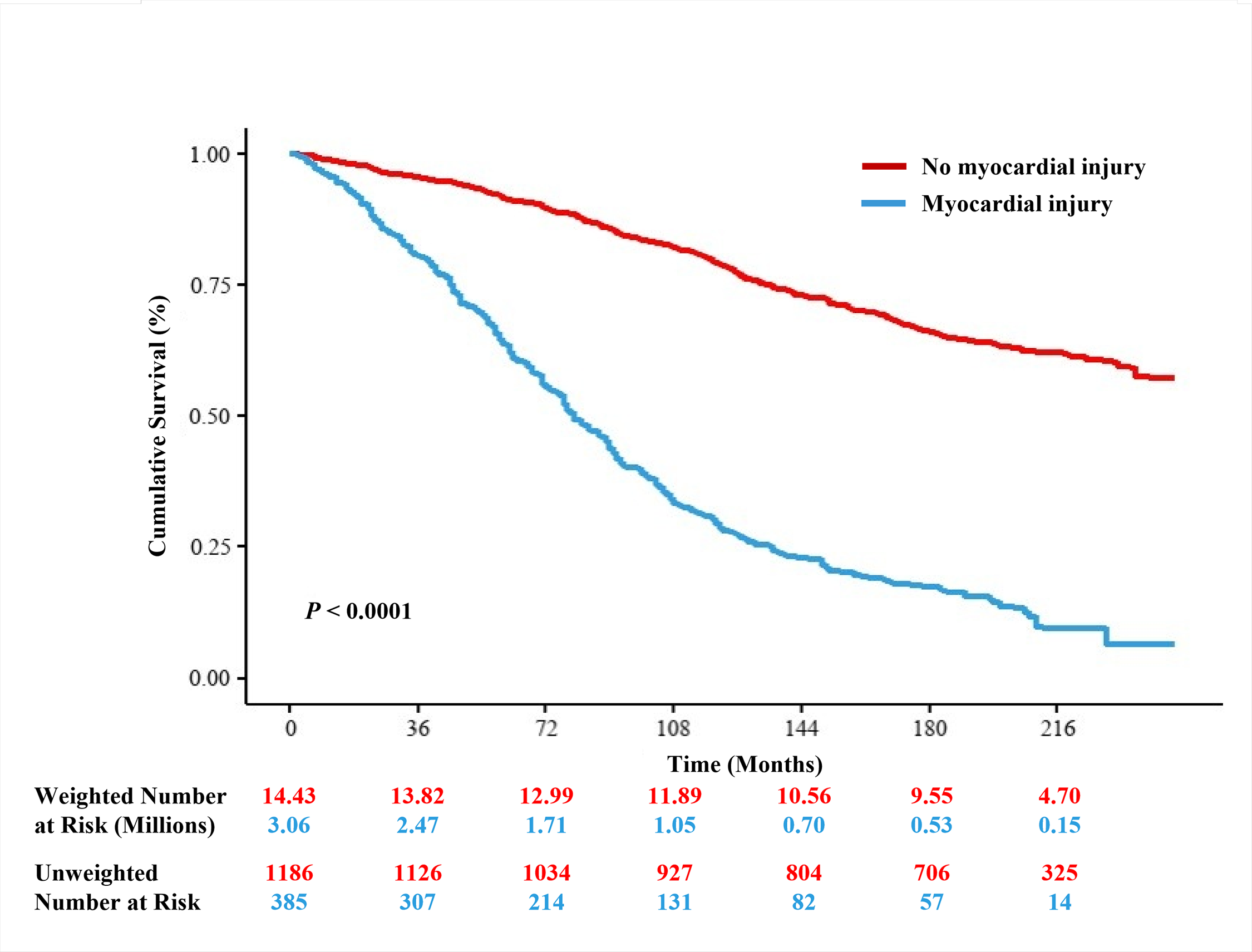

Supplement: S6 Fig — (TIF) [file pone.0354873.s013.tif]

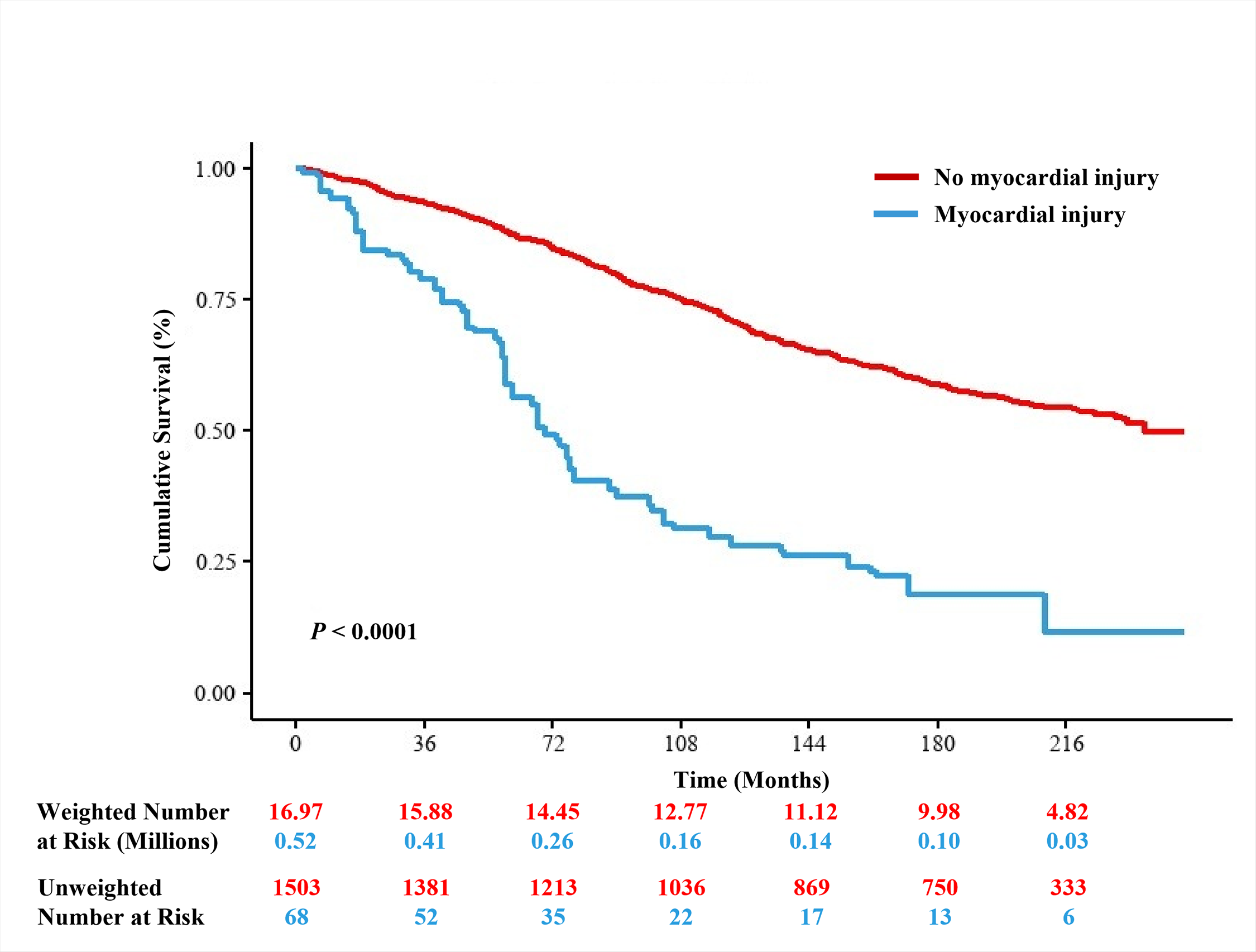

Supplement: S7 Fig — (TIF) [file pone.0354873.s014.tif]

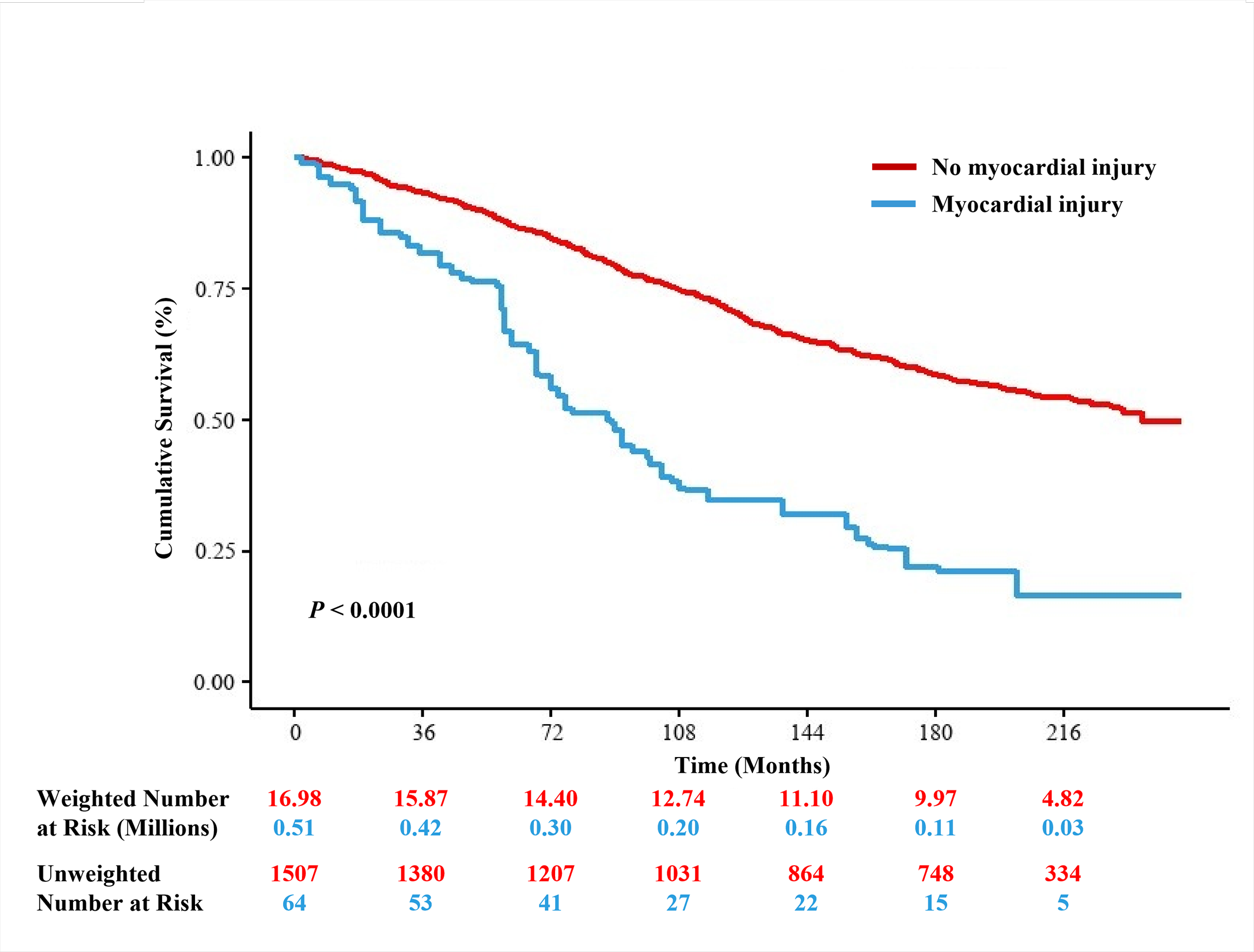

Supplement: S8 Fig — (TIF) [file pone.0354873.s015.tif]

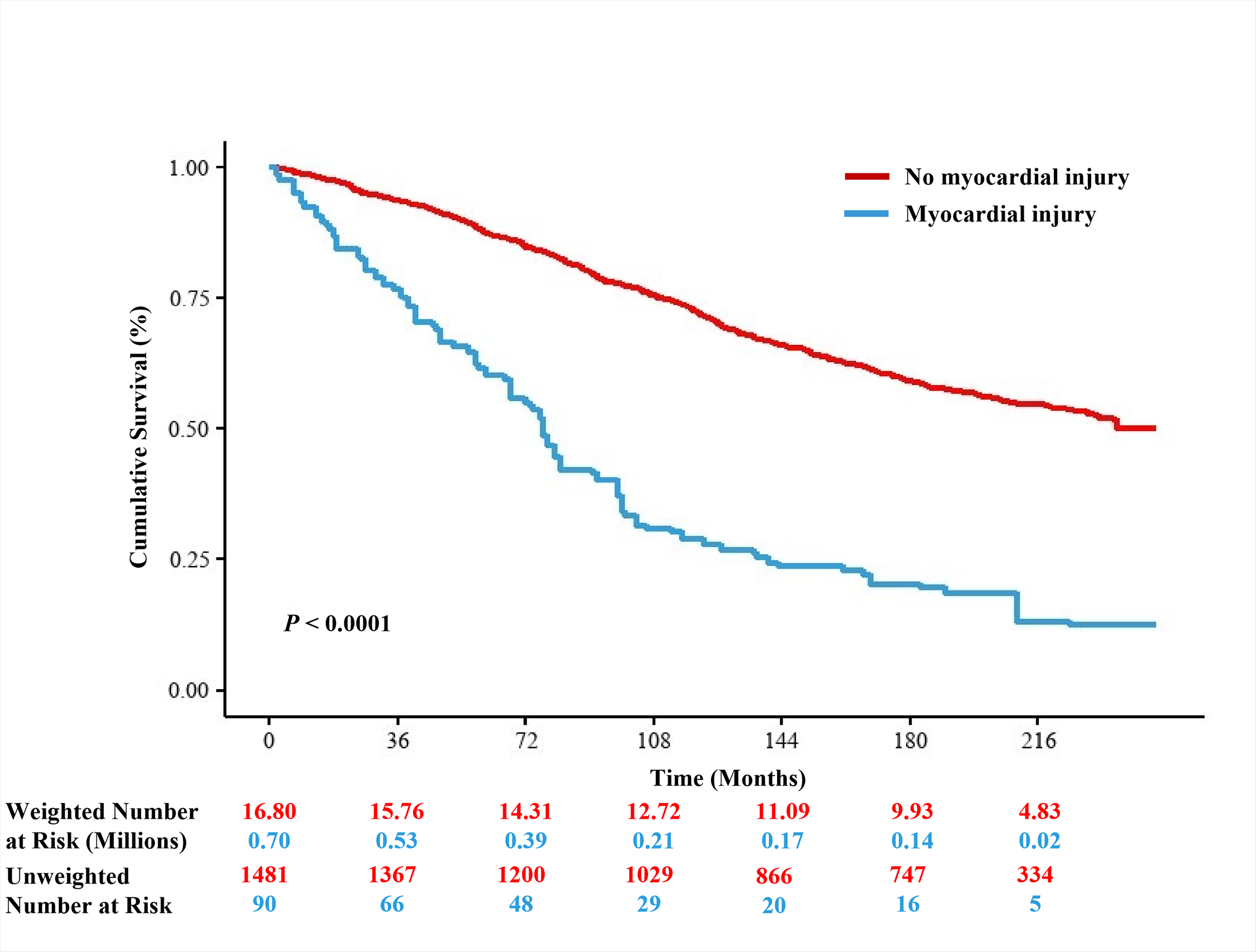

Supplement: S9 Fig — (TIF) [file pone.0354873.s016.tif]
